# An Elevated FIB-4 Score Is Associated with an Increased Incidence of Depression among Outpatients in Germany

**DOI:** 10.3390/jcm11082214

**Published:** 2022-04-15

**Authors:** David Schöler, Karel Kostev, Münevver Demir, Mark Luedde, Marcel Konrad, Tom Luedde, Christoph Roderburg, Sven H. Loosen

**Affiliations:** 1 Clinic for Gastroenterology, Hepatology and Infectious Diseases, University Hospital Düsseldorf, Medical Faculty of Heinrich Heine University Düsseldorf, Moorenstrasse 5, 40225 Düsseldorf, Germany; david.schoeler@hhu.de (D.S.); tom.luedde@med.uni-duesseldorf.de (T.L.); 2 Epidemiology, IQVIA, 60549 Frankfurt, Germany; karel.kostev@iqvia.com; 3 Clinic for Hepatology and Gastroenterology, Charité University Medical Center, Augustenburger Platz 1, 13353 Berlin, Germany; muenevver.demir@charite.de; 4 KGP Bremerhaven, 27574 Bremerhaven, Germany; mark.luedde@web.de; 5 FOM University of Applied Sciences for Economics and Management, 60549 Frankfurt am Main, Germany; konrad.marcel@web.de

**Keywords:** FIB-4, depression, mood disorders, outpatient screening

## Abstract

Background: Liver disease and depression are known to be closely associated. Non-invasive tests (NIT), such as the FIB-4 score, have been recommended by different guidelines to rule out advanced fibrosis and to stratify the risk of liver-related outcomes in patients with chronic liver diseases. However, the predictive value of an elevated FIB-4 score regarding the development of depression and/or anxiety disorders among the general population is unknown. Methods: By using the Disease Analyzer database (IQVIA), which compiles diagnoses and laboratory values as well as basic medical and demographic data of patients followed in general practices in Germany, we identified 370,756 patients with available lab values for FIB-4 score calculation between 2005 and 2019. Patients with an FIB-4 score < 2 were matched 1:1 to patients with an FIB-4 index ≥ 2 by age, sex and yearly consultation frequency. Results: In regression analysis, the incidence rate ratio (IRR) of depression was significantly higher among patients with an FIB-4 score ≥ 2.0 compared to patients with a lower FIB-4 score <2.0 (IRR: 1.12, *p* < 0.001). This association was significant for both female (IRR: 1.10, *p* = 0.004) and male (IRR: 1.15, *p* < 0.001) patients and strongest in the age groups ≤50 years (IRR: 1.42, *p* < 0.001) and 51-60 years (IRR: 1.34, *p* < 0.001). There was no significant association between an elevated FIB-4 score ≥ 2.0 and the incidence of depression among patients aged 60 years and older. There was no significant increase in the IRR of anxiety disorders for patients with high or low FIB-4 scores. Conclusion: Our study suggests a previously unknown association between an elevated FIB-4 score and an increased incidence of depression. This finding suggests that the FIB-4 score is not only a valuable tool for the prediction of liver-specific endpoints but also may be of relevance for the prediction of extrahepatic comorbidities, which in turn may argue for clinical screening programs in patients with an elevated FIB-4.

## 1. Introduction

Depression and liver disease are known to be closely associated [1]. This is attributed to multiple factors, such as increased alcohol consumption in patients with depression [2], and to hepatotoxic side effects of psychotropic drugs [3]. About every third patient with liver disease develops depression at one point in his or her life [4] and every third patient with depressive disorder develops an alcohol disorder [1]. In liver disease, inflammatory processes, cytokines, and an altered intestinal microbiome presumably contribute to the development of depression [5,6,7]. Recently, in a rat model of chronic stress, it has been shown that, after fecal microbiota transplant, the gut barrier integrity was broken, subsequently leading to liver disease and an increased inflammatory cytokine expression, with higher astrocyte activation, indicating an inflammatory process in the brain [8]. Several studies point towards a microbiota-brain-dysfunction as a potential contributor to mental disorders [9,10]. However, the underlying pathomechanisms are largely unknown. 

The FIB-4 score, which is calculated based on patients’ ages, AST/ALT serum levels and the platelet count, was initially developed as a noninvasive test to predict liver fibrosis in patients with HIV/HCV coinfection [11]. It is simple to calculate, and its parameters are usually part of a basic laboratory blood test. The FIB-4 score can also be used to predict fibrosis in patients with nonalcoholic fatty liver disease (NAFLD) [12]. Currently, it is unknown whether its application in the general population could help identify people at increased risk for depression. Its use could potentially help to enable screening measures, and a diagnosis at an early stage of the disease. Besides having high morbidity [13] and mortality [14], depression is a leading cause of disability worldwide and is of high socio-economic relevance. We therefore used the Disease Analyzer database (IQVIA) to calculate the FIB-4 score in a cohort of 370,756 patients from 924 outpatient practices in Germany and to evaluate its relevance as a potential indicator for the development of depression.

## 2. Materials and Methods

### 2.1. Database

This study was based on data from the Disease Analyzer database (IQVIA), which contains drug prescriptions, diagnoses, and basic medical and demographic data obtained directly and in anonymous format from computer systems used in the practices of general practitioners and specialists [15]. The database covers approximately 3% of all outpatient practices in Germany. Diagnoses (according to International Classification of Diseases, 10th revision (ICD-10)), prescriptions (according to Anatomical Therapeutic Chemical (ATC) Classification system), and the quality of reported data are being monitored by IQVIA. In Germany, the sampling methods used to select physicians’ practices are appropriate for obtaining a representative database of general and specialized practices. It has previously been shown that the panel of practices included in the Disease Analyzer database is representative of general and specialized practices in Germany [15]. Finally, this database has already been used in previous studies focusing on the FIB-4 index [16] and psychiatric diseases [17,18].

### 2.2. Study Population

**Figure 1:**
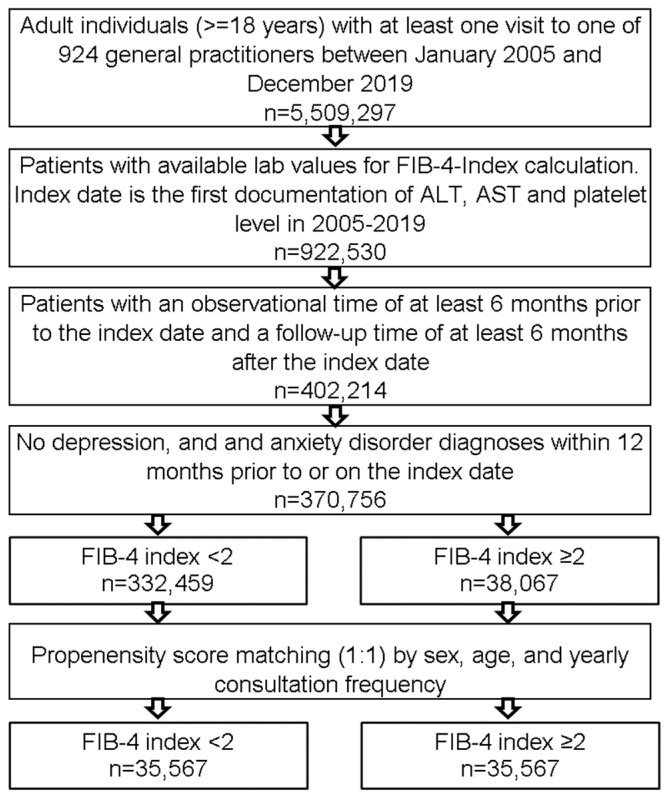
Selection of study patients.

This retrospective cohort study included adult patients (≥18 years) in 924 general practices in Germany with available lab values for the FIB-4 index calculation between January 2005 and December 2019. Index date was the first documentation of ALT, AST, and platelet level in this time period (Figure 1). Further inclusion criteria included an observation time of at least six months prior to the index date and a follow-up time of at least six months after the index date. Patients with depression (ICD-10: F32, F33) or anxiety disorder (ICD-10: F41) documented within 12 months prior to or on the index date were excluded. 

The FIB-4 index was calculated using the formula Age (yr) × AST [U/L]/(PLT [10(9)/L] × ALT[U/L](1/2)). Each patient included in the study has an average of 3.2 FIB-4 index values. The FIB-4 index was calculated per patient for the whole follow-up time.

Patients with average FIB-4 indices of <1.0, <1.3, <1.7, and <2.0 were matched to patients with FIB-4 indices of ≥1.0, ≥1.3, ≥1.7, and ≥2.0, respectively. Greedy nearest-neighbor propensity score matching (1:1) based on sex, age, Charlson Comorbidity Index [19], and yearly consultation frequency was performed. This matching was necessary due to very strong age, sex, and comorbidity differences between patients with lower and higher average FIB-4 index values.

### 2.3. Study Outcomes and Statistical Analyses

The main outcome of the study was the incidence of depression and anxiety disorder as a function of an average FIB-4 index calculated per patient for the whole follow-up time (<1.0 versus ≥1.0, <1.3 versus ≥1.3, <1.7 versus ≥1.7, and <2.0 versus ≥2.0). Univariate Poisson regression models were conducted to study the ratio of two incidence rates (FIB-4 index ≥1.0 vs. <1.0, ≥1.3 vs. <1.3, ≥1.7 vs. <1.7, and ≥2.0 vs. <2.0). To counteract the problem of multiple comparisons, *p*-values <0.01 were considered statistically significant. As only FIB-4 index ≥2.0 vs. <2.0 was significantly associated with an increased incidence of depression, detailed analyses were further performed for these cohorts. Differences in the sample characteristics between those with an FIB-4 index <2 and those with an FIB-4 index ≥2 were tested using McNemar tests for categorical variables and paired Wilcoxon tests for continuous variables. Incidence as the number of cases per 1000 patient-years was calculated. Regression analyses were performed separately for women and men and four age groups (age ≤ 50, age 51–60, age 61–70, age 71–80, and age > 80). Analyses were carried out using SAS version 9.4 (SAS institute, Cary, NC, USA).

## 3. Results

### 3.1. Preliminary Analyses

We first aimed at establishing a clinically useful FIB-4 cut-off value that allows for the identification of patients with an increased incidence of depression and/or anxiety disorders. In univariate Poisson regression models, there were no significant associations between an FIB-4 index ≥1.0, ≥1.3, and ≥1.7 and the incident of depression or anxiety disorder within 10 years from the index date. However, we observed a highly increased incidence of depression among patients with an FIB-4 score ≥2.0 compared to patients with a score <2 (incident rate ratio (IRR): 1.12 (95% CI: 1.06–1.17, *p* < 0.001). In contrast, there was no association between an FIB-4 score ≥2.0 and anxiety disorders. The results of the preliminary analyses are shown in Table 1.

### 3.2. Basic Characteristics of the Study Sample

Based on the preliminary analysis, we next established a cohort of 35,567 patients with an FIB-4 index ≥2.0 that were matched to a cohort of equal size with an FIB-4 index < 2.0 by sex, age, Charlson Comorbidity index (CCI), and yearly consultation frequency. More than 95% of patients with an FIB-4 index ≥2 had FIB-4 values between 2.0 and 3.0. The mean age of the study cohorts was 71.8 (FIB-4 < 2)/71.9 (FIB-4 ≥ 2) years with a mean CCI of 2.9/2.8, and 44.8/44.6% of patients were female. There were no significant differences with respect to age, sex, or CCI between the two cohorts. The basic characteristics of the study cohort are displayed in Table 2.

### 3.3. A FIB-4 Score ≥2.0 Is Associated with an Increased Incidence of Depression

Within ten years from the index date, the incidence of depression was significantly higher among patients with an FIB-4 score ≥ 2.0. As such, these patients had an incidence of depression per 1000 person-years of 24.6 compared to only 22.0 in patients with an FIB-4 score < 2. In regression analysis, we observed a significantly higher depression incidence rate ratio (IRR) of depression in patients with a FIB-4 score ≥ 2.0 compared to patients with an FIB-4 score < 2.0 (IRR: 1.12, *p* < 0.001, Table 3). This association was significant for both female (IRR: 1.10, *p* = 0.004) and male (IRR: 1.15, *p* < 0.001, Table 3) patients. The strongest difference was observed in the age groups ≤ 50 years (IRR: 1.42, *p* < 0.001) and 51–60 years (IRR: 1.34, *p* < 0.001). There was no significant association between an elevated FIB-4 score ≥ 2.0 and the incidence of depression among patients aged 60 years and above (Table 3).

### 3.4. Association of FIB-4 Index ≥2.0 and Anxiety Disorders

Based on the promising results showing an association between an elevated FIB-4 score ≥ 2.0 and the incidence of depression, we finally evaluated if the incidence of anxiety disorders is likewise increased among these patients. However, in regression analyses, there was no significant increase in the incidence rate ratio of anxiety disorders between patients with an FIB-4 score </≥ 2.0 for any analyzed subgroup (Table 3). Of note, patients with an FIB-4 score ≥ 2.0 aged 51 to 60 years showed a trend towards a higher incidence of anxiety disorders (Table 3).

## 4. Discussion

According to the WHO, depression is a major contributor to the overall global burden of disease. About 280 million people in the world have depression [20]. More than 75% of people with depression in low- and middle-income countries receive no treatment, and still, around 63% in high-income countries receive no treatment [21]. Strikingly, about half of patients with depression are unrecognized in primary care settings [22], which accounts in part for the high number of untreated patients and necessitates improved diagnostics in this field. Most especially, the early detection of depression is difficult [23], as presentations could be masked by irritability or physical complaints [24]. Recently, it has been shown in a randomized controlled trial that early detection and treatment of depression in primary care has a positive effect on response and remission rate [25], underlining the importance of early depression detection. Additionally, efforts have been made to integrate artificial intelligence in the early detection of depression [26,27].

By using the Disease Analyzer database (IQVIA, Frankfurt, Germany), which compiles diagnoses, laboratory values, and basic medical and demographic data for over 7.5 million patients followed in general practices in Germany in the study time period [15,16], we identified a total of 370,756 patients with available lab values for FIB-4 score calculation. Importantly, this cohort was shown to be representative for the general population in Germany.

It is known that risk stratification of patients with advanced fibrosis can be improved by automatically calculating the FIB-4 score, and its use as a predictive parameter in the development of liver cancer has recently been validated in a large cohort of 29,999 patients with nonalcoholic fatty liver disease [16], confirming the European Association for the Study of the Liver recommendation of implementing the FIB-4 score as a non-invasive fibrosis test in populations at risk of liver fibrosis [28].

Both, male and female patients with a FIB-4 score ≥ 2 had a significantly increased risk of developing depression within 10 years from the index date. Notably, the development of depression in patients with an FIB-4 score ≥ 2 was strongest in patients ≤ 60 years. Interestingly, a higher depression IRR was already observed with higher FIB-4 score cut-off values, i.e., in the groups with an FIB-4 cutoff of 1.3 and 1.7 (Table 1), though not significantly. In addition, anxiety disorder IRR was also higher in the FIB-4 ≥ 2 group in the age group 51–60 years but without reaching significance. Thus, in the outpatient setting, a focus could be laid on patients at a higher risk of depression development, lowering the threshold for further work-up, e.g., by depression questionnaires (PHQ-8 etc. [29]) and referral to a specialist. The automatic calculation in most routine examinations makes it a practical parameter, facilitating the decision for further psychiatric work-up.

Although the FIB-4 score is currently used in the area of liver disease, our results suggest that this score can also be used as an important tool to screen for extrahepatic comorbidities, specifically in the early detection of depression. A potential explanation for this finding could lie in the gut–liver–brain axis, which is attracting increasing attention in the recent years [30,31]. Interestingly, a disturbed inflammatory response in liver and brain was attributed to *PCSK9* methylation in chronic alcohol use [32]. It is known that patients with mood and anxiety-related disorders exhibit evidence of elevated inflammatory markers [33]. Inflammatory signals are thought to play an important role in the development of depression and anxiety disorder, e.g., the cytokines IL-1β, IL-6, and TNFα [34]. A number of risk factors associated with increased inflammation have been linked to the development of depression, many of them being amenable to therapeutic interventions [34]. Furthermore, a change in microbiota is considered to be crucial in the effects on the gut-liver-brain axis [35]. These studies suggest that further investigations in mouse models with fecal microbiota transplant and systematic analysis of inflammatory mechanisms in the brain could unravel knowledge in the gut–liver–brain axis, with potential therapeutic implications [36,37].

Research regarding anxiety disorders and the gut–liver–brain axis is still in its infancy [38], and the pathomechanisms compared to depression development are to a large extent unknown. However, a unique microbiome signature was found to be associated with major depression and general anxiety disorder, e.g., *Faecalibacterium* in the human gut was significantly lower in a general anxiety disorder relative to a major depressive disorder [39]. Therefore, different specific pathomechanisms in the emergence of depression vs. anxiety disorder could be responsible for our observation that the anxiety disorder IRR was not significantly changed in the FIB-4 ≥ 2 group.

The aforementioned studies and our data point towards a higher vulnerability of depression development in the age group 51–60 years. A “window of vulnerability” for the development of depression has been observed in women who have passed menopause [40,41]. Additionally, in the general population, a mean age of 49 was found for the onset of depressive disorders [42]. Patients < 51 years of age accounted for only a small fraction (5.7%) in our study cohort (Table 2). Of note, all patients included in this study were previously not diagnosed with depression, indicating that an elevated FIB-4 score preceded the development of depression. As the Charlson Comorbidity index was similar in both groups with an FIB-4 score ≥ 2.0 and < 2.0 (Table 2), it seems unlikely that the higher depression IRR in the FIB ≥ 2 group is attributable to a higher comorbidity but specific for the FIB-4 components age, platelet count, and AST/ALT.

This study has several limitations: The database did not include data on patients’ symptoms, with regard to potential reactive depressions, e.g., because of an acute liver disease; potential alleviations of depression in the time course, of a potentially self-limiting acute liver disease, were not analysed. Therefore, it will be necessary to perform a detailed analysis of patients who show an improvement in the FIB-4 score over time, versus those whose FIB-4 score remains elevated. Furthermore, we had no data on socioeconomic factors (e.g., education, income, and social support) and lifestyle-related risk factors (e.g., alcohol/drug use, physical activity, and nutritional status). In addition, thyroid function, which is known to be associated with psychiatric disorders [43], is not taken into account in the Charlson Comorbidity index. Further limitations of our study concern the study design, which is based on retrospective database analyses. The ICD-10 coding system was used, which sometimes leads to misclassification/undercoding of certain diagnoses. In addition, the lab values needed for FIB-4 scores were available in only around 17% of patients, which could cause selection bias. Of note, the IQVIA database does not provide mortality data, which is also important with respect to suicidality rates in people with depression. ICD-10 codes for other neuropsychiatric diseases, e.g., bipolar disorder, dysthymia, or other anxiety disorders, were not included in the exclusion criteria of this study. This might lead to a selection bias because other neuropsychiatric diseases, such as bipolar disorder, dysthymia, and other anxiety disorders might lead to similar symptoms as in depression and anxiety disorder. Finally, the existence of liver disease in patients with an FIB-4 score > 2 was not analysed, which needs to be investigated in future studies.

Considering a lower cut-off for the FIB-4 score in younger patients to rule out fibrosis [44], more studies are needed to unravel the predictive value of higher FIB-4 scores in an age-dependent manner. Future studies are needed to elucidate the temporal connection between index date and the development of depression and to analyze the factors that have the greatest influence on depression development; it is commonly known that major depression has multifactorial causes, including social, psychological, and biological aspects [45]. Analyses of genetic risk factors by use of genome wide studies [46] and inflammatory pathways [47] underline the complexity of the disease. Of note, as outlined before, more and more studies shed light on the connection between mental health and the gut microbiome [6], the latter being an important potential link between liver disease and depression. In summary, our study suggests that the FIB-4 score is not only relevant for hepatic diseases but also for extrahepatic endpoints, such as depression and other psychiatric diseases. However, it will be relevant to unravel potential crosslinks of hepatic and extrahepatic diseases using combined analyses in the future.

## Figures and Tables

**Table 1:** Association between the FIB-4 index and incidence of depression and anxiety disorder within 10 years from index date in patients followed in general practices in Germany depending on different FIB-4 index cut-off values (univariable Poisson regression models).

FIB-4 Index Cut-Off Values	Incidence Rate Ratio (95% CI)	*p*-Value
Depression		
≥1.0 vs. <1.0 (n = 141,436)	0.99 (0.96–1.02)	0.641
≥1.3 vs. <1.3 (n = 128.902)	1.03 (1.00–1.07)	0.076
≥1.7 vs. <1.7 (n = 97,608)	1.04 (1.00–1.08)	0.039
≥2.0 vs. <2.0 (n = 71.134)	1.12 (1.06–1.17)	<0.001
Anxiety disorder		
≥1.0 vs. <1.0 (n = 141,436)	0.94 (0.88–0.99)	0.031
≥1.3 vs. <1.3 (n = 128.902)	1.00 (0.94–1.07)	0.968
≥1.7 vs. <1.7 (n = 97,608)	1.10 (1.01–1.19)	0.021
≥2.0 vs. <2.0 (n = 71.134)	1.07 (0.98–1.18)	0.138

**Table 2:** Basic characteristics of the study sample after 1:1 propensity score matching by sex, age, and yearly consultation frequency.

Variable	Proportion of Patients with FIB-4 < 2 (%) N = 35,567	Proportion of Patients with FIB-4 ≥ 2 (%) N = 35,567	*p*-Value
Age (Mean, SD)	71.8 (11.8)	71.9 (11.8)	0.628
Age ≤ 50	5.7	5.7	0.802
Age 51–60	10.8	10.9	
Age 61–70	22.0	21.8	
Age 71–80	37.9	37.7	
Age > 80	23.7	23.9	
Women	44.8	44.6	0.702
Men	55.2	55.4	
Charlson comorbidity Index (Mean, SD)	2.9 (2.9)	2.8 (2.9)	0.571
Yearly consultation frequency during the follow-up time (Mean, SD)	5.1 (5.9)	5.0 (5.5)	0.159

Proportions of patients given in %, unless otherwise indicated. SD: standard deviation.

**Table 3:** Association between an FIB-4 index ≥ 2 and the incidence of depression and anxiety disorder within 10 years from the index date in patients followed in general practices in Germany stratified by age and sex (univariable Poisson regression models).

	Patients with FIB-4 <2; Incidence per 1000 Person-Years	Patients with FIB-4 ≥2; Incidence per 1000 Person-Years	Incidence Rate Ratio (FIB-4-Index ≥2.0 vs. <2.0) (95% CI)	*p*-Value
Depression				
Total	22.0	24.6	1.12 (1.06–1.17)	<0.001
Age ≤ 50	22.3	31.7	1.42 (1.19–1.71)	<0.001
Age 51–60	23.2	31.0	1.34 (1.18–1.51)	<0.001
Age 61–70	18.0	18.9	1.05 (0.94–1.16)	0.386
Age 71–80	21.7	22.9	1.05 (0.98–1.14)	0.178
Age >80	28.3	29.9	1.06 (0.95–1.17)	0.293
Women	29.1	32.1	1.10 (1.03–1.17)	0.004
Men	16.9	19.4	1.15 (1.07–1.23)	<0.001
Anxiety disorder				
Total	5.4	5.8	1.07 (0.98–1.18)	0.138
Age ≤ 50	6.9	8.0	1.15 (0.83–1.60)	0.390
Age 51–60	5.1	6.6	1.31 (1.00–1.71)	0.046
Age 61–70	4.4	4.9	1.14 (0.93–1.39)	0.224
Age 71–80	5.5	5.6	1.02 (0.88–1.20)	0.764
Age >80	6.7	6.3	0.94 (0.76–1.16)	0.576
Women	7.0	7.6	1.09 (0.96–1.24)	0.182
Men	4.3	4.6	1.07 (0.93–1.23)	0.371

## Data Availability

The data that support the findings of this study are available on request from the corresponding authors.
